# Fasting total ghrelin levels are increased in patients with adolescent idiopathic scoliosis

**DOI:** 10.1186/s13013-015-0054-7

**Published:** 2015-11-30

**Authors:** Jérôme Sales de Gauzy, Isabelle Gennero, Olivier Delrous, Jean-Pierre Salles, Benoit Lepage, Franck Accadbled

**Affiliations:** Pediatric Orthopaedics Unit, Children Hospital, CHU de Toulouse, France; Biomechanics Laboratory, Paul-Sabatier University, Toulouse, France; INSERM Unit 1043, Physiopathology Center of Toulouse Purpan (CTPT), Paul-Sabatier University, Toulouse, France; Biochemistry Laboratory, Institut Fédératif de Biologie, University Hospital Center, CHU de Toulouse, France; Endocrine and Bone Diseases Unit, Children Hospital, University Hospital Center, CHU de Toulouse, France; Pediatric Clinical Investigation Center, Children Hospital, University Hospital Center, CHU de Toulouse, France; Department of Epidemiology, University Hospital Center, Paul-Sabatier University, CHU de Toulouse, France

**Keywords:** Ghrelin, Hormone, Adolescent idiopathic scoliosis (AIS), Girl, Spine

## Abstract

**Background:**

A control study was designed to investigate circulating Ghrelin levels in adolescent girls with adolescent idiopathic scoliosis (AIS) and controls. Eating behavioral disorders, endocrine disorders, abnormal growth pattern and osteopenia have been well documented in AIS. Ghrelin is an orexigenic hormone produced by the stomach which reflects body weight changes and stimulates growth hormone secretion. Recently, it has been shown to be associated with bone metabolism and eating behavior. However, the circulating levels of ghrelin have never been evaluated in AIS patients.

**Methods:**

Forty nine AIS girls and 15 controls were included. Anthropometric parameters and fasting circulating total ghrelin were measured. Curve severity was evaluated in AIS girls. The relationships between ghrelin and age, body weight, height, body mass index (BMI), BMI Z-score and corrected anthropometric parameters were analyzed in AIS girls and controls.

**Results:**

There was no significant difference in body weight, height, BMI or BMI Z-score between AIS and controls. Serum ghrelin level was 1.8 fold higher in AIS girls than in controls. Elevation of ghrelin levels remained significant when corrected BMI or corrected BMI Z-score were considered. Unlike in controls, positive correlations were found between ghrelin and age in AIS girls with a gradual increase of circulating ghrelin with age.

**Conclusions:**

We have observed significantly higher circulating ghrelin levels in AIS than in controls with a positive correlation with age. This pilot-study suggests that ghrelin signaling might play a role in the initiation or development of AIS. Further studies are needed to validate theses results.

## Introduction

Adolescent Idiopathic Scoliosis (AIS) is characterized by a three-dimensional spinal deformity which occurs during prepubertal and pubertal growth. Etiology of AIS remains unknown. Hypotheses include genetic, skeletal, muscular, biochemical, biomechanical, neurohormonal and environmental factors [1].

A frequent characteristic of girls with AIS is that they have significantly lower body weight and lower BMI compared to healthy counterparts during the early and middle pubertal stages [2–4]. Alborghetti et al. demonstrated correlation between AIS and eating disorders [5]. Incidence of AIS patients below undernourishment degree 1 or even within the margins of anorexia nervosa (AN) is above the general population [6–8].

Ghrelin is a peptide hormone which plays a major role in hunger stimulation. Ghrelin is secreted primarily from the stomach. Plasma ghrelin levels are directly related to food intake episodes. Ghrelin is secreted in a pulsated manner as its level increases before the onset of meal, during fasting, and decreases after a meal. This pulsatile secretion of ghrelin suggested that ghrelin may act as a signal for meal initiation [9]. Inverse correlations were reported between ghrelin and BMI or fat mass [10, 11]. Studies on eating behaviors suggest that ghrelin is not only dependent on body fat mass, but also influenced by the nutritional status [9].

Therefore, considering the low BMI and eating disorders in AIS, we hypothesized that ghrelin anomaly may be involved in scoliosis and that AIS patients may have abnormal circulating ghrelin levels. The aim of our study was to compare ghrelin serum level in patients with and without AIS.

## Materials and Methods

AIS patients were recruited in the Pediatric Orthopedic Unit of our hospital. Signed informed consent was obtained from the parents of all subjects. Approval was granted by the local IRB of Toulouse University Hospital (ID-RCB:2009-A011011-56, granted 01/19/2010). The control group was represented by scoliosis free children admitted in the department of surgery for various minor elective procedures (curly toes correction, physiodesis, or solitary osteochondroma resection), with clinical examination and blood sampling routinely performed before anesthesia. All subjects included in the current study met the following criteria: no evidence of any endocrine diseases, history of eating disorders or steroid intake. In total, 49 AIS (12 to 17.5 years old) and 15 control Caucasian girls (12 to 17 years old) were included.

For scoliotic patients, corrected height was computed by adjusting trunk loss using Bjure formula: LogY = 0.011X - 0.177, where Y is the loss of trunk height (cm) caused by the spinal deformity, and X is the Cobb angle of the primary curve. Weight was measured on a digital scale in the fasting state to the nearest 0.1 kg in normal indoor clothing and barefoot. BMI was calculated by dividing weight (kg) by height squared (m^2^) and corrected BMI (cBMI) by dividing weight (kg) by corrected height squared (m^2^). BMI and cBMI were then expressed in Z-score according to the reference data for the French population (21).

Overnight fasting blood was obtained for ghrelin level in both AIS patients and controls. Serum samples were obtained after centrifugation and stored at −80 °C until assay. At the end of the study, total ghrelin was measured using a commercial radioimmunoassay (Phoenix Pharmaceuticals, Belmont, CA) with a detection limit at 2 pg/ml. The intra and interassay coefficients of variation were 7.2 and 9.9 %, respectively. Data were analyzed using STATA SE v11.0. Data reported as means ± SD. P < 0.05 was considered significant. We used Student’s t test or Wilcoxon rank sum test to compare the two samples. Univariate and multiple regression analyses were performed to determine predictors of hormonal secretion. Square root transformation was performed to approximate a normal distribution for the ghrelin data. Pearson or Spearman’s rank correlations between circulating ghrelin levels and growth-related parameters (age, weight, height, BMI, corrected height and corrected BMI) were estimated and compared in AIS and controls.

## Results

Clinical features of adolescents with AIS and controls are summarized in Table 1. Wilcoxon test analysis showed that when compared to controls, height of AIS patients was not significantly different, but corrected height calculated with the Bjure formula showed a 5.1 centimeter difference, AIS patients being taller than controls (163.2 ± 7.3 *vs* 158.1 ± 10.1). This difference was close to significance (*p = 0.09*). The other anthropometric parameters were not significantly different, therefore the two groups were comparable. All 49 patients had at least one severe spinal curve at the time of the blood sample, with an average Cobb angle of 56.1 ± 13.2 degrees for the main curve. Twenty five patients had a second curve of 54.2 ± 12.9 degrees.

**Table 1:** Physical characteristics and total plasma Ghrelin level in AIS patients and controls

	AIS (*n*=49)	Controls (n=15)	*p*
Age (yr)	14.3 ± 1.4	13.9 ± 1.6	*0.23 †*
Weight (kg)	48.3± 8.0	46.9 ± 10.8	*0.98 †*
Height	158.8 ± 7.3	158.1 ± 10.1	*0.97 †*
cHeight (cm)	163.2 ± 7.3	-	*0.09 †*
BMI (kg/m^2^)	19.1 ± 2.6	18.5 ± 2.5	*0.42 §*
cBMI (kg/m^2^)	18.1 ± 2.5	-	*0.61 §*
BMI Z	−0.02 ± 1.14	−0.16 ± 1.01	*0.67 §*
cBMI Z	−0.46 ± 1.23	-	*0.39 §*
Main curve Cobb angle (°)	56.1 ± 13.2	-	
2nd curve Cobb angle (*n*=25)	54.2 ± 12.9	-	
Ghrelin (pg/mL)	261.9 ± 120.3	146.1 ± 59.2	*<0.001 †*

Values are shown as means ± SD. In controls, Corrected height (cHeight), corrected BMI (cBMI), corrected BMI Z-score (cBMI Z) were equal to height, BMI, BMI Z, of them respectively. † Wilcoxon non parametric test. § Student’s t-test

We found a significantly 1.8 fold higher mean level of total serum ghrelin in AIS group compared to the control population (261.9 ± 120.3 vs 146.1 ± 59.2; *p < 0.001*).

Correlations between fasting ghrelin concentrations and age or anthropometric measurements are exhibited in Table 2. In the control group, ghrelin revealed a strong negative correlation with age (*r* = −0.60; *p = 0.02*). Conversely, in the AIS group, the correlation was positive (*r* = 0.19; *p = 0.20*) and this difference of evolution between AIS and controls was significant (*p = 0.006*). Weight, height, corrected height, BMI and corrected BMI were also moderately inversely correlated with ghrelin levels in the control group, while they were close to zero in the AIS group, but these correlations did not reach statistical significance. In both groups, circulating ghrelin correlated neither with the BMI Z-score nor with the corrected BMI Z-score.

**Table 2:** Correlations between circulating Ghrelin level and growth related parameters in AIS patients and controls (Ghrelin concentrations were normalized with a square root transformation)

	AIS *(n=49)*		Control *(n=15)*		
	R	*p*	R	*p*	*p†*
Age (years)	0.19	*0.20*	−0.60	*0.02*	*0.006*
Weight (kg)	0.01	*0.97*	−0.31	*0.26*	*0.31*
Height (cm)	−0.04	*0.79*	−0.35	*0.21*	*0.32*
cHeight (cm)	−0.08	*0.59*	−0.35	*0.21*	*0.38*
BMI (kg/m^2^)	0.02	*0.91*	−0.27	*0.33*	*0.36*
cBMI (kg/m^2^)	0.04	*0.80*	−0.27	*0.33*	*0.33*
BMI Z-score	−0.08	*0.60*	−0.07	*0.81*	*0.98*
cBMI Z-score	−0.04	*0.79*	−0.07	*0.81*	*0.94*

R represents Pearson’s correlation coefficients for normative data. † Correlation coefficients comparison after Fisher’s z transformation. Corrected height (cHeight), corrected BMI (cBMI), corrected BMI Z-score (cBMI Z)

## Discussion

Previous studies have reported lower BMI and eating disorders in population of AIS. Siu et al. reported significantly lower body weights and BMI in AIS girls compared with controls between the age of 12 and 15 [4]. Ramirez et al. investigated BMI and body composition in 27 girls with AIS and compared them to general population [6]. They found a real alteration of body composition in AIS. The BMI, Fatfreemass index and Fatmass index were lower than in the general population. Smith et al. studied weight, height and BMI in 44 young women with AIS. Compared with normative data, patients from AIS group were significantly lighter and had significantly lower BMI scores, 25 % of the series had BMI scores within the definition of anorexia. Barrios et al. showed a progressive decrease of BMI with age in 52 AIS girls [2]. In their study, a total of 21.2 % of AIS girls had a BMI below 17.5 which is the threshold for anorexia, while the incidence in the control group was only 3.3 %.

Several studies have highlighted a significantly larger proportion of individuals with eating disorders in AIS, which severity was correlated with the presence of AN [5, 8]. However, Zaina et al., using the Eat-26 questionnaire, showed a low prevalence of eating disorders in AIS female patients [12]. They concluded that even if low BMI individuals are more frequent in AIS, this cannot be considered a sign of eating disorder. They attributed low BMI in AIS to hormonal alterations also involved in the onset of scoliosis. Qiu et al. have suggested that leptin plays a significant role in the pathogenesis of AIS [13]. Leptin level is low in AIS. It has the effect of both reducing the fat mass and altering bone formation.

Our study suggests that inadequate secretion of ghrelin may be involved in AIS. A 1.8 fold higher ghrelin level was observed in the AIS group, even after controlling confounding factors.

Elevated ghrelin levels have been reported in AN compared with normal-weighted controls [14–16]. The increase of plasma ghrelin levels in AN seems paradoxical in light of the restrained food intake of these patients and suggests an adaptive response [17]. A state of ghrelin resistance has been suspected in AN [15, 17] but also in Prader Willi Syndrome (PWS) [18], a condition frequently associated with scoliosis [19]. Inadequate secretion of ghrelin may be hypothesized in AIS, associated, alike in AN and PWS, to some degree of ghrelin resistance.

In addition to its role as the “hunger hormone”, ghrelin plays many other roles [20]. Therefore, elevated ghrelin or resistance may explain other abnormal findings in AIS such as delayed puberty and bone demineralization.

A late menarche has been reported to be associated with higher prevalence of AIS [21]. Furthermore, girls with severe scoliosis experienced later menarche than those with mild scoliosis [22–24]. In the general population, a progressive decline in circulating ghrelin level with age is observed, a phenomenon supposed to enable puberty onset. Therefore, persistent high ghrelin level may contribute to delay puberty in AIS patients.

Generalized reduced bone mass and osteopenia in both axial and peripheral skeletons have been reported in AIS [25–27]. Scoliosis causes osteopenia and osteoporosis among girls while their siblings with normal spine remain with normal bone mass [7].There is a decreased osteogenic differentiation of mesenchymal stem cell and reduced bone mineral density in AIS patients [28]. This can be due to some ghrelin resistance, because ghrelin increases proliferation and differentiation of osteoblasts in vitro and bone mineral density (BMD) in vivo [29]. A variety of hormones, neurotransmitters, and biologically active substances control the functions of living bodies via specific receptors located in cell membranes. Many of these receptors mediate the transmission of intracellular signals by activating guanine nucleotide-binding proteins (G proteins) to which the receptor is coupled. Such receptors are generically referred to as G protein-coupled receptors (“GPCR”s) [30]. Ghrelin secretion is stimulated by several Gs-coupled receptors and inhibited by many Gi-coupled and Gq receptors [31]. Given that a Gi-signalling dysfunction has been reported in AIS [32], it is conceivable that Gi protein hypofunctionality in AIS could contribute to the elevation of plasma ghrelin levels in AIS patients.

We acknowledge several weaknesses to this study. Magnitude (Cobb angle) of scoliosis was high, up to 50°. Nutritional as well as menstrual statuses were missing. The small sample size as well as the unbalanced size of the control group both decreased the power of the study. Nonetheless, the difference of ghrelin levels between AIS and control groups was large enough to be statistically significant. We measured only total ghrelin which represents both acylated and deacylated forms. However, only Acylated ghrelin stimulates appetite [33]. There is no population based reference data of ghrelin level in the specific age group.

Nevertheless, these results open perspectives of further research and if confirmed, they may have some applications in screening and management of AIS. Chronic central administration of ghrelin increases bone mass through a mechanism that is independent of body weight, suggesting that ghrelin may have a bone anabolic effect through the central nervous system [34]. Furthermore, ghrelin administrated intravenously to human also leads to appetite increase and food intake stimulation [35].

## Conclusion

This is the first pilot-study investigating total fasting serum ghrelin levels in AIS. The key finding was the 1.8 fold higher ghrelin levels observed in the AIS group, even after controlling for confounding factors, suggesting that ghrelin signaling might play a role in the pathogenesis of AIS. Results need to be validated by further studies including larger and more representative populations, with a calculation of the appropriate sample size beforehand.

## Abbreviations

AIS: Adolescent idiopathic scoliosis
GPCR: G protein-coupled receptors
